# The Ubiquitin Proteasome System in Ischemic and Dilated Cardiomyopathy

**DOI:** 10.3390/ijms20246354

**Published:** 2019-12-17

**Authors:** Sabine Spänig, Kristina Kellermann, Maja-Theresa Dieterlen, Thilo Noack, Sven Lehmann, Michael A. Borger, Jens Garbade, Yaron D. Barac, Fabian Emrich

**Affiliations:** 1 Department of Cardiac Surgery, Heart Center Leipzig, University Leipzig, 04289 Leipzig, Germany; kristina_kellermann@web.de (K.K.); mdieterlen@web.de (M.-T.D.); Thilo.Noack@helios-gesundheit.de (T.N.); Sven.Lehmann@medizin.uni-leipzig.de (S.L.); Michael.Borger@helios-gesundheit.de (M.A.B.); Jens.Garbade@medizin.uni-leipzig.de (J.G.); fabian.emrich@t-online.de (F.E.); 2 The Cardiothoracic Surgery Department, Rabin Medical Center, Sackler Faculty of Medicine, Tel Aviv University, Tel Aviv 6997801, Israel; yaronbar@icloud.com

**Keywords:** ubiquitin proteasome system, cardiac remodeling, ischemic cardiomyopathy, dilated cardiomyopathy, E3 ligases, protein ubiquitination

## Abstract

Dilated (DCM) and ischemic cardiomyopathies (ICM) are associated with cardiac remodeling, where the ubiquitin–proteasome system (UPS) holds a central role. Little is known about the UPS and its alterations in patients suffering from DCM or ICM. The aim of this study is to characterize the UPS activity in human heart tissue from cardiomyopathy patients. Myocardial tissue from ICM (*n* = 23), DCM (*n* = 28), and control (*n* = 14) patients were used to quantify ubiquitinylated proteins, E3-ubiquitin-ligases muscle-atrophy-F-box (MAFbx)/atrogin-1, muscle-RING-finger-1 (MuRF1), and eukaryotic-translation-initiation-factor-4E (eIF4E), by Western blot. Furthermore, the proteasomal chymotrypsin-like and trypsin-like peptidase activities were determined fluorometrically. Enzyme activity of NAD(P)H oxidase was assessed as an index of reactive oxygen species production. The chymotrypsin- (*p* = 0.71) and caspase-like proteasomal activity (*p* = 0.93) was similar between the groups. Trypsin-like proteasomal activity was lower in ICM (0.78 ± 0.11 µU/mg) compared to DCM (1.06 ± 0.08 µU/mg) and control (1.00 ± 0.06 µU/mg; *p* = 0.06) samples. Decreased ubiquitin expression in both cardiomyopathy groups (ICM vs. control: *p* < 0.001; DCM vs. control: *p* < 0.001), as well as less ubiquitin-positive deposits in ICM-damaged tissue (ICM: 4.19% ± 0.60%, control: 6.28% ± 0.40%, *p* = 0.022), were detected. E3-ligase MuRF1 protein expression (*p* = 0.62), NADPH-oxidase activity (*p* = 0.63), and AIF-positive cells (*p* = 0.50). Statistical trends were detected for reduced MAFbx protein expression in the DCM-group (*p* = 0.07). Different levels of UPS components, E3 ligases, and UPS activation markers were observed in myocardial tissue from patients affected by DCM and ICM, suggesting differential involvement of the UPS in the underlying pathologies.

## 1. Introduction

Cardiac remodeling is a complex process, involving cardiomyocyte growth and death, fibrosis, inflammation, and electrophysiological remodeling, and may be accompanied by a reduced ejection fraction and arrhythmias [1]. Cardiomyopathies, particularly ischemic cardiomyopathy (ICM) and dilative cardiomyopathy (DCM), may induce left ventricular remodeling. ICM occurs as a result of reduced cardiac perfusion caused by coronary artery disease or myocardial infarction. Hypoxia is the main cause of ischemia and is accompanied by a lack of oxygen supply to cells [2,3]. Ischemia in myocardial tissue leads to a dramatic increase in oxygen radicals, which induce the development of reactive oxygen species (ROS) due to NAD(P)H oxidase and can cause considerable damage to cell components [2,4]. Heart muscle tissue is replaced by connective tissue, which leads to both impaired left-ventricular function and dilatation of the affected ventricle [5]. The etiology of DCM is often unknown and might be multifactorial. Potential causes can include infectious, toxic, metabolic, immunologic, neuromuscular, endocrine, or valvular factors, among others [6]. Besides acquired forms of DCM, 20–48% of all DCM cases are familial. Familial DCM has a very heterogeneous genotype that can be either autosomal dominant or x-linked. DCM is characterized by ventricular dilatation and a limited systolic function (ejection fraction <45%), and leads to progressive heart failure (HF), decreased left-ventricular contraction, ventricular and supraventricular arrhythmias, and sudden cardiac death [7]. Both ICM and DCM can cause substantial cardiac dysfunction and are thus considered the most common causes of heart transplantation and/or mechanical support [8].

An extensive list of studies has established the direct role of the ubiquitin–proteasome system (UPS) in cardiac hypertrophy and HF, mediated by its impact on cardiac apoptosis, cell mass, quality control in the sarcomere, β-adrenergic signaling, cell excitability, and conductance. Its protein degradation activities are responsible for clearance of over 90% of all misfolded, oxidized, or otherwise damaged mammalian proteins, thereby preventing intrinsic toxicity [9]. The UPS consists of the proteasome, ubiquitin, the ubiquitination machinery, and deubiquitinases (DUBs). A functional proteasome holds different proteolytic activities and binding sites for substrates and ubiquitin [1,10,11]. Ubiquitin is an 8.5 kDa, highly conserved, globular protein which is attached to the amino group of its target protein [12,13]. The ubiquitin molecule contains seven lysine residues, where the specific lysine residue tagging the target protein determines the protein’s fate. An enzymatic cascade, consisting of an E1 ubiquitin-activating- enzyme (E1), an E2 ubiquitin-conjugating enzyme (E2), and an E3 ubiquitin ligase (E3), drives ubiquitination of the target proteins. Throughout the catalytic cascade, the enzyme multiplicity increases dramatically. In the human proteome, there are two E1, approximately 40 E2, and over 600 E3 enzymes [14,15]. Substrates can be either mono-, multi-, or poly-ubiquitinated. While single ubiquitin can be attached to the NH2-terminus of proteins, the vast majority of ubiquitin is linked to the free NH2 of internal lysine residues of the targeted proteins [16]. Multi-ubiquitinated means that more substrate lysines are bound with a single ubiquitin, and poly-ubiquitinated means that ubiquitin can be conjugated as a polymer by the sequential addition of further ubiquitins to each other through ubiquitin lysines [17]. Poly-ubiquitination can occur on lysine 48 (K48), which leads to proteasomal degradation, or lysine 63 (K63), that channels proteins to degradation by autophagy [18,19]. In vitro, a minimum of four ubiquitins bind to a single lysine residue, but in vivo, over 20 ubiquitins per lysine can be observed.

To maintain protein homeostasis, the two main pathways of protein degradation UPS and autophagy are essential. While UPS is responsible for degrading short-lived proteins, autophagy is responsible for degrading long-lived proteins [20]. UPS regulation mechanisms and induction of autophagy are related to each other through sharing similar substrate recognition sites and their mode of action [21]. Ubiquitination plays an important role for the cross talk of the two degradative systems. Ubiquitin chains bind selectively by the ubiquitin-binding domains of adaptors, which link the substrates to the proteasome or autophagic vacuoles [21]. Besides K48 and K63 linkages, K11 and K29 linkages induce proteasomal degradation, whereas K6 linkages trigger autophagic degradation [22].

Deubiquitinases (DUBs) remove ubiquitin from target proteins and, hence, regulate proteasomal degradation [23]. Although the UPS bears a central role in cardiac remodeling and is thought to be a suitable target to inhibit or reverse the remodeling processes in the heart, little is known about the changes it undergoes in the pathologically remodeled human heart. The present study compared the status of UPS components in the myocardium of healthy versus patients with severe ICM and DCM.

## 2. Results

Western blot analysis for the translation marker eIF4E showed comparable protein expression in myocardial tissue obtained from ICM, DCM, and control patients (control: 0.89 ± 0.08, ICM: 1.02 ± 0.11, DCM: 0.98 ± 0.08; *p* = 0.67), suggesting that the level of protein synthesis was comparable in all groups.

### 2.1. Proteasome Activity

**Figure 1:**
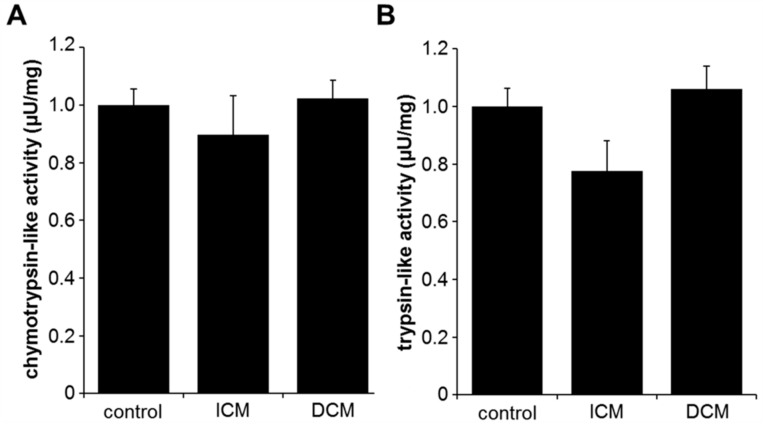
Proteasomal activity of the chymotrypsin-like (**A**) and trypsin-like (**B**) catalytic domains in myocardial tissue from control (*n* = 11), ischemic cardiomyopathy (ICM) (*n* = 19), and dilative cardiomyopathy (DCM) (*n* = 22) patients. Results are expressed as mean ± SEM.

Proteasomal activity was analyzed by quantification of the activity of its various catalytic domains (Figure 1). No significant differences in the activity of the chymotrypsin-like (control: 1.00 ± 0.06 µU/mg, ICM: 0.90 ± 0.14 µU/mg, DCM: 1.02 ± 0.07 µU/mg, *p* = 0.71) (Figure 1A) or caspase-like (control: 0.06 ± 0.85 µU/mg, ICM: 0.18 ± 0.73 µU/mg, DCM: 0.19 ± 0.70 µU/mg, *p* = 0.93) domains of the proteasome were observed in myocardial tissue from ICM, DCM, and control patients. The trypsin-like domain showed a statistical trend towards reduced proteasome activity in ICM tissue (0.78 ± 0.11 µU/mg), compared to DCM (1.06 ± 0.08 µU/mg) and control (1.00 ± 0.06 µU/mg, *p* = 0.06) tissue (Figure 1B).

### 2.2. Myocardial Protein Ubiquitination

**Figure 2:**
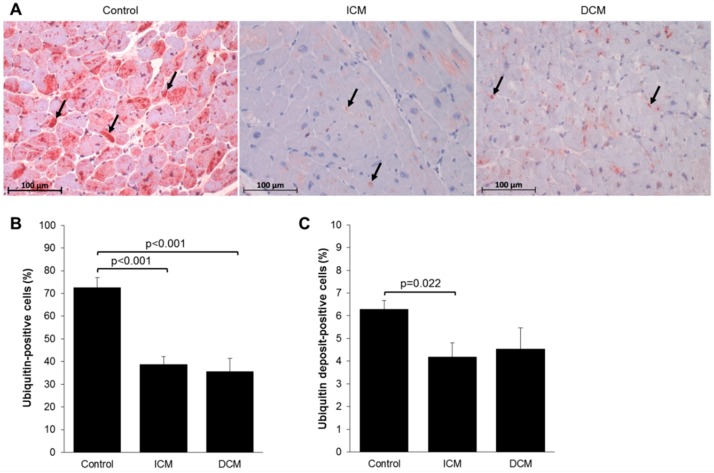
(**A**) Immunohistochemical staining for ubiquitin on sectioned myocardium from control (*n* = 10), ICM (*n* = 11), and DCM (*n* = 10) patients. Arrows denote ubiquitin deposits. Quantification of ubiquitin staining of (**B**) ubiquitin-positive cells and (**C**) ubiquitin deposit-positive cells. Results are expressed as mean ± SEM.

Analysis of ubiquitin-stained myocardial tissue sections (Figure 2) showed a significant reduction in the percentage of ubiquitin-positive cells in ICM (38.81 ± 3.35%, *p* < 0.001) and DCM (35.69 ± 5.76%, *p* < 0.001) tissue compared to control tissue (72.57 ± 4.36%) (Figure 2B). Furthermore, the percentage of cells with intracellular ubiquitin deposits was significantly lower in ICM (4.19 ± 0.60%, ICM vs. control *p* = 0.022) compared to control tissue (6.28 ± 0.40%) (Figure 2C). A smaller percentage as compared to control was also seen in DCM tissue (4.54 ± 0.93%, DCM vs. control *p* = 0.226), but the difference was not significant.

### 2.3. Expression of E3 Ligases

**Figure 3:**
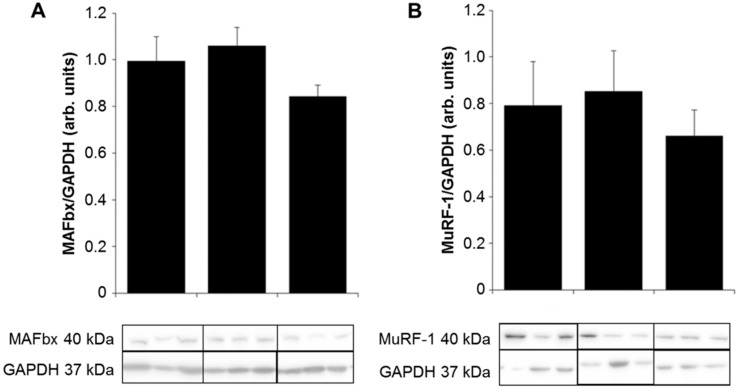
Densitometric analysis of Western blots for (**A**) MAFbx and (**B**) MuRF-1 expression in myocardial tissue lysates from control (*n* = 11), ICM (*n* = 19), and DCM (*n* = 22) patients. GAPDH was used as loading control. Results are expressed as mean ± SEM.

Western blot analyses for the myocardial E3 ligases muscle atrophy F-box (MAFbx) and muscle ring-finger protein-1 (MuRF1) showed comparable expression levels in the three groups (Figure 3), although for MAFbx there was a tendency towards decreased expression in DCM (0.84 ± 0.05) tissue compared to ICM (1.06 ± 0.03) and control tissue (0.99 ± 0.10, *p* = 0.07) (Figure 3A). MuRF1 protein expression was not statistically different between the three groups (control: 0.79 ± 0.19, ICM: 0.85 ± 0.18, DCM: 0.66 ± 0.11; *p* = 0.62) (Figure 3B).

### 2.4. Oxidative Stress and Apoptosis

**Figure 4:**
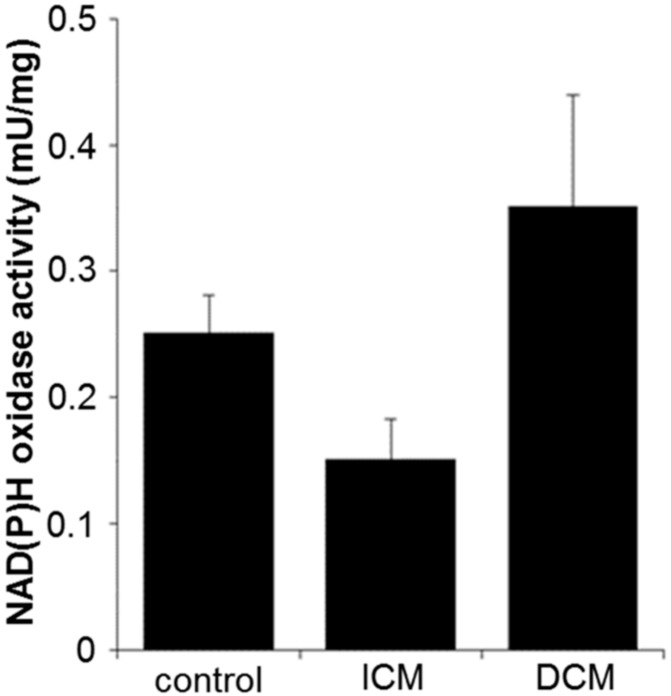
Quantification of NAD(P)H oxidase activity per milligram protein in myocardial tissue from control (*n* = 11), ICM (*n* = 19), and DCM (*n* = 22) patients. Results are expressed as mean ± SEM.

**Figure 5:**
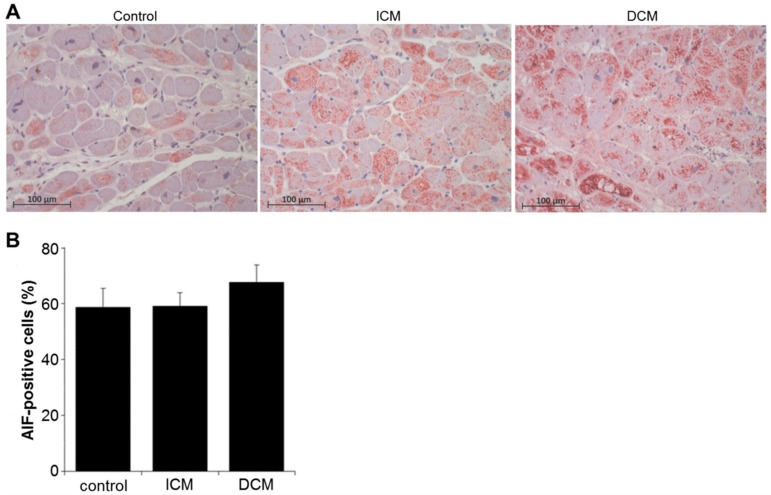
Immunohistochemical staining (**A**) and quantitative analysis (**B**) for apoptosis-inducing factor (AIF) on sectioned myocardium from control (*n* = 10), ICM (*n* = 11), and DCM (*n* = 10) patients. Results are expressed as mean ± SEM.

Compared to control tissue (0.25 ± 0.03 mU/mg), the NAD(P)H oxidase activity decreased by trend in ICM tissue (0.15 ± 0.03 mU/mg, *p* = 0.09) and was unchanged in DCM tissue (0.35 ± 0.09 mU/mg, *p* = 0.63) (Figure 4). Because NAD(P)H oxidase activity influences oxidative stress, which, in turn, may lead to apoptosis, the percentage of apoptotic cells was quantified by staining for the apoptosis-inducing factor (AIF). However, the expression of the early apoptosis marker AIF did not differ between the groups (control: 58.80 ± 6.74%, ICM: 59.18 ± 4.84%, DCM: 67.68 ± 6.23%; *p* = 0.50) (Figure 5B).

## 3. Discussion

The present study compared UPS expression and activity in myocardial tissue obtained from DCM patients, ICM patients, and from unaffected cardiac tissue. Minimal but consistent differences in proteasome activity were noted between the groups, with ICM tissue exhibiting a trend for a loss of trypsin-like proteasomal activity compared to the other tissue samples. This observation agrees with the report by Predmore and colleagues, demonstrating reduced proteasomal activity in failing hearts caused by oxidative modification of proteasome subunits [24]. The study compared samples collected from failing hearts in comparison to a control group consisting of non-HF tissue [24]. In our study, exclusively end-stage HF tissue was used, and sought out differences between ICM- and DCM-induced damage.

It has been hypothesized that protein aggregates in failing hearts become increasingly cross-linked, preventing them from degradation by the proteasome, thus inhibiting proteasomal function [25,26,27]. Kostin et al. detected deposits of ubiquitin-positive aggregates in human DCM [28]. In our study, we detected a decreased percentage of ubiquitin-positive cells and of cells with ubiquitin deposits in both ICM and DCM tissue.

Investigations of the third component of the UPS, the ubiquitination machinery, and the muscle-specific E3 ubiquitin ligases, revealed a tendency for reduced MAFbx levels, but no change in E3 ligase MuRF-1 protein levels in DCM tissue. MAFbx acts as a fundamental regulator of myocardial remodeling by regulating ubiquitin-dependent degradation of the calcium-activated phosphatase calcineurin, which is required for pathological hypertrophy, and thereby inhibits pathological hypertrophy [29]. MAFbx is also found in skeletal muscle as an inhibitor of physiological, adaptive hypertrophy, because it enhances the activity of FoxO1 and FoxO3a fork head transcription factors, which activate genes responsible for muscle wasting [30,31]. MAFbx mediates the polyubiquitination of FoxO transcription factors by K48- and K63-linked ubiquitin chains [32].

MuRF-1, a sarcomere-associated protein, is known to inhibit pathological hypertrophy [9]. Conraads et al. speculated that MuRF-1 and MAFbx permit hypertrophy in areas of low expression from the site of infarction [33]. In the present study, no significant differences in MuRF-1 protein expression were observed between DCM, ICM, and control tissue. Unaltered MuRF-1 protein expression in pressure overload myocardium in comparison to healthy controls has been documented, while other E3 ligases such as Cbl, E6AP, Mdm2, and cIAP were found to be altered [34]. In contrast, Willis et al. detected increased MuRF-1 protein expression in patients with end-stage ischemic heart disease [35]. While the present study compared end-stage ICM and DCM tissue to healthy samples, the possibility of the latter being adaptive hypertrophic tissue due to aortic valve stenosis cannot be ignored. Changes in the UPS observed in this tissue might have already been pathologic [36]. This represents one of the major problems when investigating cardiomyopathies, since at the time of tissue collection, we cannot be sure whether the changes in UPS activity observed are causative or a compensatory response to the changes in the myocardium.

The UPS influences apoptosis-regulating proteins [37]. The relationship between the UPS and apoptosis was investigated by Tsukamoto et al., who reported depressed proteasome activities before the onset of cardiac dysfunction, accompanied by apoptosis of cardiomyocytes. In turn, cardiomyocytes apoptosis contributed to the progression of cardiac dysfunction in pressure-overloaded hearts [38]. In the present study, the number of AIF-positive cells did not significantly differ between ICM, DCM, and control tissue, suggesting that apoptosis was not altered in the cardiomyopathic tissue. This, again, might be due to the fact that we assessed end-stage HF tissue and not the various developmental stages of cardiomyopathy.

Myocardial ischemia results in increased oxygen radical production, which, in turn, induces damage of cellular components [39]. In the present study, we found reduced NAD(P)H oxidase activity in ICM tissue and increased activity in DCM tissue.

In summary, we investigated the status of the UPS in ICM and DCM end-stage HF patients in comparison to its status in healthy controls. While chymotrypsin- and caspase-like proteasomal activity was comparable between the groups, trypsin-like proteasomal activity was decreased by trend in ICM samples. In addition, decreased ubiquitin expression was noted in both cardiomyopathies, and fewer less ubiquitin-positive deposits were observed in ICM-damaged tissue. There were no differences observed in E3-ligase MuRF1 protein expression, NADPH-oxidase activity, or AIF-positive cell counts. Statistical trends for reduced MAFbx protein expression was detected in the DCM group. Taken together, we found evidence of UPS alterations in end-stage HF tissue samples, but further research, possibly requiring samples from the different stages of HF progression, is needed to determine its mechanistic relevance to the development of cardiomyopathies.

### Limitations

The main limitation of the present study was probably the control tissue, which was obtained from Morrow resections during aortic valve replacement. Although for the control patients no hypertrophic cardiomyopathy (HOCM) was diagnosed, one might assume that resection occurred due to a sub valvular flow restriction, which is usually the case in some type of hypertrophy. Therefore, the control tissue might also be pathologically altered and not truly healthy. Still, as the control patients all had a preserved ejection fraction and as it is difficult to obtain truly healthy ventricular tissue for research purposes, in our opinion, this serves as acceptable “control”. Also, comparing samples from different locations within the heart (septal tissue and LV apex) is unfortunate, but in our opinion, sufficient for this study. Another limitation of this study is that we were looking at end-stage cardiomyopathy tissue from patients during LVAD implantation. At this time point, one cannot be sure whether the observed changes in the myocardium are developmental for the pathology or a compensatory mechanism. Still, as the character of this study is rather preliminary in characterizing the UPS in the remodeled heart and looking for potential new targets, we believe this study can serve as basis for further research in this field.

## 4. Materials and Methods

### 4.1. Sample Collection and Preparation

Tissues were collected at the Leipzig Heart Center in Germany, following approval by the local IRB committee and patient consent (Approval No: 240/16ek; Date: 27 October 2016). Human myocardial tissue was obtained from the cardiac apex after left ventricular assist device (LVAD) implantation or from Morrow resections (septal tissue of patients with aortic stenosis, but with no diagnosed hypertrophic cardiomyopathy) that were used as a control group. Myocardial tissue from ICM (*n* = 23), DCM (*n* = 28), and control (*n* = 14) patients was prepared, dissected, and stored (1) at −80 °C for protein and enzyme analysis and (2) in 70% ethanol after fixing for 24 h in 4% formalin–PBS, for immunohistological analyses. Tissue samples for immunohistochemistry were embedded in paraffin.

### 4.2. Quantification of Protein Expression

Frozen tissue samples were homogenized in lysis buffer (50 mM Tris, 1 mM EDTA, 150 mM NaCl, 1% Nonidet-P40, 0.25% deoxycholic acid sodium salt, 1× Protease and Phosphatase Inhibitor Cocktail (Thermo Scientific, Dreieich, Germany), 1% phenylmethylsulfonyl fluoride (PMSF)), sonicated in an ultrasound ice water bath, and centrifuged at 16,000 rcf at 4 °C for 10 min. Following protein extraction, the protein content was determined using a bicinchoninic acid (BCA) assay for colorimetric detection (Pierce BCA Protein Assay Kit, Thermo Fisher Scientific, Waltham, MA, USA), by measuring the absorbance at 560 nm on a spectrophotometer. Western blot analyses were performed using specific primary antibodies against MAFbx (generated in rabbit against the CYPRKEQYGDTLQL peptide sequence, Eurogentex, Seraing, Belgium), MURF1 (Abcam), eIF4E (Santa Cruz Biotechnology, Dallas, TX, USA), and glyceraldehyde 3-phosphate dehydrogenase (GAPDH) (Hytest, Turku, Finland). Each gel contained a balanced amount of samples from all three groups. Horseradish peroxidase-conjugated secondary antibodies (Sigma, St. Louis, MO, USA) were used. Bands were visualized by enzymatic chemiluminescence (SuperSignal West Dura Extended Duration Substrate, Thermo Fisher Scientific). Densitometry was performed using Fusion VisionCapt v16 software (VWR, Radnor, PA, USA).

### 4.3. Quantification of Proteasome Activity

For quantification of the proteasome activity, the standard protocol of Bowen T. S. et al. was used [40]. Frozen tissue samples were homogenized in proteasome lysis buffer (10 mM Tris, 1 mM EDTA, 2 mM ATP, 20% glycerin, 4 mM dithiothreitol), sonicated, and centrifuged at 16,000 rcf at 4 °C for 10 min. To ensure that only the proteasomal activity was measured, the samples were treated with the proteasomal inhibitor MG132 (Sigma) in parallel as a negative control. Chymotrypsin-like, trypsin-like, and peptidylglutamyl-peptide hydrolyzing activities of the proteasome were analyzed using the fluorogenic peptides Suc-Leu-Leu-Val-Tyr-7-amino-4-methylcoumarin (LLVY-AMC) (Sigma), benzonyl-Val-Gly-Arg-4-methylcoumarin (Bz-VGR-AMC) (Enzo Life Sciences, Exeter, UK), and benzyloxy-carbonyl-Leu-Leu-Glu-methylcoumarylamide (Z-LLE-AMC) (Sigma). Protein (20 µg) was incubated with reaction buffer (0.05 mol/L Tris-HCl, pH 8.0, 0.5 mmol/L EDTA) and 40 µmol/L of one of three fluorogenic peptides. The reaction was inhibited by addition of the proteasome inhibitor MG132. The kinetic fluorescence was detected by a spectrophotometer (Tecansafire, Tecan, Crailsheim, Germany) at 380 nm excitatory (Ex) and 440 nm emission (Em) wavelengths. The enzymatic activity was calculated from a calibration curve of free 7-amino-4-methylcoumarine (AMC) (Sigma).

### 4.4. Quantification of Activity of A Reactive Oxygen Species-Producing Enzyme

Frozen tissue samples were homogenized in proteasome lysis buffer, sonicated, and centrifuged at 16,000 rcf, at 4 °C for 10 min. The activity of nicotinamide adenine dinucleotide phosphate (NAD(P)H) oxidase (Roth, Arlesheim, Switzerland), a potent reactive oxygen species (ROS)-producing enzyme, was measured photometrically via a UV/Vis spectrometer (Lamda 20, Perkin Elmer, Rodgau, Germany) at 550 nm. After addition of 4 mM cytochrome *c* and 100 µmol/L NADH to the tissue homogenate, the proportion of cytochrome *c* reduction in the presence NADH was determined. ROS responsible for the cytochrome *c* reduction, derives predominantly from the NAD(P)H oxidase.

### 4.5. Immunohistological Analysis

Fixed and paraffin-embedded myocardial tissues were cross-sectioned into 3 µm sections. For staining, sections were deparaffinized in xylene, dehydrated in ethanol (100%, 95%, 70%, 50%), and rehydrated in distilled water. After washing in tris-buffered saline (TBS), sections were permeabilized in 0.01 M sodium citrate, in a microwave (800 Watts), for 30 min, and allowed to cool. Peroxidase activity was blocked with 0.3% H_2_O_2_/TBS, followed by blocking of nonspecific sites with 2% bovine serum albumin in TBS. For observation of apoptosis and ubiquitin distribution, specimens were incubated with primary antibodies against anti-apoptosis-inducing factor (AIF; 1:80, Santa Cruz) and multi-ubiquitin (1:50, Biozol, Uppsala, Sweden), overnight at 4 °C, followed by incubation with rabbit-anti mouse IgG peroxidase-conjugated secondary antibody (1:400, Sigma). After washing, tissue sections were stained with the 3-amino-9-ethylcarbazole (AEC) Substrate Chromogen kit (Dako, Glostrup, Denmark). For nuclear staining, Mayer’s Hematoxylin (Dr. K. Hollborn *&* Söhne, Leipzig, Germany) was used. The fluorescence images of AIF and ubiquitin myocardial sections were captured (×400) by a single blinded investigator using a Axioplan 2 microscope and AxioVision software (both Carl Zeiss, Jena, Germany).

### 4.6. Statistical Analysis

Statistical analysis was performed with SPSS 23.0 (IBM Inc., Armonk, NY, USA). Differences between the groups were analyzed by one-way analysis of variance (ANOVA), which included the Levene’s test to assess the equality of variances. In the case of variance inequality, the Welch test was performed for global testing and the Dunnett’s T3 method for the post-hoc algorithms. In the case of equal variances, data were analyzed with the Scheffe post-hoc test. Data are represented as mean ± standard deviation, unless stated otherwise. *p*-values of significance were *p* ≤ 0.05 and trends *p* ≤ 0.10.
